# 2023 International African Swine Fever Workshop: Critical Issues That Need to Be Addressed for ASF Control

**DOI:** 10.3390/v16010004

**Published:** 2023-12-19

**Authors:** Lihua Wang, Llilianne Ganges, Linda K. Dixon, Zhigao Bu, Dongming Zhao, Quang Lam Truong, Juergen A. Richt, Meilin Jin, Christopher L. Netherton, Charaf Benarafa, Artur Summerfield, Changjiang Weng, Guiqing Peng, Ana L. Reis, Jun Han, Mary-Louise Penrith, Yupeng Mo, Zhipeng Su, Dang Vu Hoang, Roman M. Pogranichniy, David-Adrian Balaban-Oglan, Yuzhen Li, Kewen Wang, Xuepeng Cai, Jishu Shi

**Affiliations:** 1 Center on Vaccine Evaluation and Alternatives for Antimicrobials, College of Veterinary Medicine, Kansas State University, Manhattan, KS 66506, USA; lihua@vet.k-state.edu (L.W.); yuzhen@vet.k-state.edu (Y.L.); 2 WOAH Reference Laboratory for Classical Swine Fever, IRTA-CReSA, 08193 Barcelona, Spain; llilianne.ganges@irta.cat; 3 IRTA, Programa de Sanitat Animal, Centre de Recerca en Sanitat Animal (CReSA), Bellaterra, 08193 Barcelona, Spain; 4 The Pirbright Institute, Ash Road, Pirbright, Woking GU24 0NF, UK; linda.dixon@pirbright.ac.uk (L.K.D.); christopher.netherton@pirbright.ac.uk (C.L.N.); ana.reis@pirbright.ac.uk (A.L.R.); 5 State Key Laboratory for Animal Disease Control and Prevention, National African Swine Fever Para-Reference Laboratory, National High Containment Facilities for Animal Diseases Control and Prevention, Harbin Veterinary Research Institute, Chinese Academy of Agricultural Sciences, Harbin 150069, China; buzhigao@caas.cn (Z.B.); zhaodongming@caas.cn (D.Z.); 6 Key Laboratory of Veterinary Biotechnology, Faculty of Veterinary Medicine, Vietnam National University of Agriculture, Hanoi 12406, Vietnam; tqlam@vnua.edu.vn; 7 Diagnostic Medicine/Pathobiology, College of Veterinary Medicine, Kansas State University, Manhattan, KS 66506, USA; jricht@ksu.edu; 8 College of Veterinary Medicine, Huazhong Agricultural University, Wuhan 430070, China; jml8328@126.com (M.J.); penggq@mail.hzau.edu.cn (G.P.); 9 Institute of Virology and Immunology IVI, Federal Department of Home Affairs, 3147 Mittelhäusern, Switzerland; charaf.benarafa@ivi.admin.ch (C.B.); artur.summerfield@unibe.ch (A.S.); 10 Department of Infectious Diseases and Pathobiology, Vetsuisse Faculty, University of Bern, Postfach, 3012 Bern, Switzerland; 11 Multidisciplinary Center for Infectious Diseases, University of Bern, Postfach, 3012 Bern, Switzerland; 12 National African Swine Fever Para-Reference Laboratory, Harbin Veterinary Research Institute, Chinese Academy of Agricultural Sciences, Harbin 150069, China; wengchangjiang@caas.cn; 13 College of Veterinary Medicine, China Agricultural University, Beijing 100107, China; hanx0158@cau.edu.cn; 14 Department of Veterinary Tropical Diseases, University of Pretoria, Hatfield 0028, South Africa; marylouise_penrith@yahoo.com; 15 Dekang Agriculture and Animal Husbandry Group, Chengdu 610225, China; 741623673@qq.com; 16 Liyuan Group, Guilin 541001, China; 17 National Institute of Veterinary Research, Hanoi 100000, Vietnam; dangvuhoang@nivr.gov.vn; 18 Veterinary Diagnostic Laboratory, Department of Diagnostic Medicine/Pathobiology, College of Veterinary Medicine, Kansas State University, Manhattan, KS 66506, USA; rmp1@vet.k-state.edu; 19 Romanian Association of Swine Veterinarians, 013821 Bucharest, Romania; adrian.balaban@fatrom.ro; 20 Faculty of Veterinary Medicine, University of Agronomic Science and Veterinary Medicine of Bucharest, 011464 Bucharest, Romania; 21 Swine Unit, Zoetis China, Beijing 102208, China; kewen.wang@zoetis.com; 22 Chinese Veterinary Drug Association, Beijing 100081, China

**Keywords:** African swine fever, workshop, platform, highlights, lessons, control, prevention

## Abstract

The 2023 International African Swine Fever Workshop (IASFW) took place in Beijing, China, on 18–20 September 2023. It was jointly organized by the U.S.-China Center for Animal Health (USCCAH) at Kansas State University (KSU) and the Chinese Veterinary Drug Association (CVDA) and sponsored by the United States Department of Agriculture Foreign Agricultural Service (USDA-FAS), Harbin Veterinary Research Institute, and Zoetis Inc. The objective of this workshop was to provide a platform for ASF researchers around the world to unite and share their knowledge and expertise on ASF control and prevention. A total of 24 outstanding ASF research scientists and experts from 10 countries attended this meeting. The workshop included presentations on current ASF research, opportunities for scientific collaboration, and discussions of lessons and experiences learned from China/Asia, Africa, and Europe. This article summarizes the meeting highlights and presents some critical issues that need to be addressed for ASF control and prevention in the future.

## 1. Introduction

African swine fever (ASF) is a contagious and severe hemorrhagic transboundary viral disease of swine (wild/feral and domestic) with up to 100% mortality rate; it causes tremendous socio-economic losses worldwide [1]. ASF has become a major problem for the global pork industry in recent years and is listed as a legally notifiable disease that must be reported to the World Organization for Animal Health (WOAH; formerly OIE) [2].

**Figure 1:**
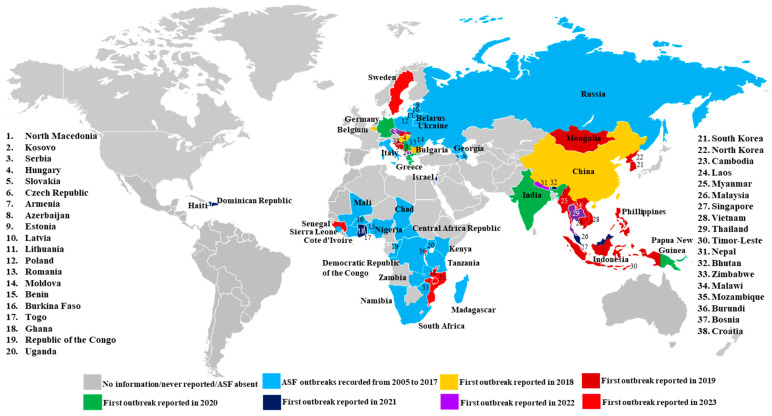
Global distribution of ASF, 2005–2023. This map is based on data from Situation reports for African swine fever of WOAH (https://www.woah.org/en/disease/african-swine-fever#ui-id-2, accessed on 10 December 2023) and Global Disease Monitoring Reports (https://www.swinehealth.org/global-disease-surveillance-reports, accessed on 10 December 2023). The names of countries with ASF are given on the map. Countries with continuing ASF outbreaks were labeled with the year when the first outbreak was reported since 2005.

ASF was first reported in Africa in 1921 and emerged for the first time in Europe in I957 [3,4]; it was eradicated from Europe by the mid-1990s, except for the Italian island of Sardinia. In 2007, ASF was detected in the Caucasus region of the Republic of Georgia and rapidly spread through the Transcaucasus region (Georgia, Azerbaijan, Armenia) and then to western Russia, followed by outbreaks in neighboring countries [5,6]. The situation changed dramatically in August 2018 when China declared an outbreak of ASF. The ASF virus spread quickly to 31 Chinese provinces within a few months. Soon after its introduction to China, ASF outbreaks occurred in many other pork-producing countries in Asia, including Bhutan, Cambodia, India, Indonesia, Laos, Malaysia, Mongolia, Myanmar, Nepal, North Korea, Singapore, South Korea, Thailand, Vietnam, and the Philippines [7,8,9,10,11,12]. In 2019/2020, the disease spread to Oceania, with ASF reported in Timor-Leste and Papua New Guinea [9,13]. In 2021, ASF was detected in the Western Hemisphere on the Caribbean Island of Hispaniola (Dominican Republic and Haiti) after a nearly 40-year absence [14,15]. Currently, ASF is widespread and endemic in sub-Saharan Africa, including parts of West Africa and the island of Sardinia, and continues to spread in Europe, Asia, the Pacific, and Caribbean regions (Figure 1).

As shown in Table 1, since January 2021, ASF has been reported in five different regions involving 50 countries, affecting over 953,000 domestic pigs and 28,000 wild boars, with more than 1,508,000 domestic pigs being culled.

**Figure 2:**
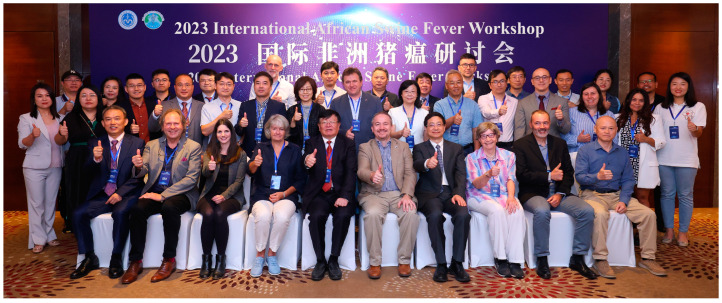
2023 International African Swine Fever Workshop (IASFW) at Beijing, China on 18–20 September 2023. Twenty-four ASF scientists from 10 countries, the Director of USCCAH at KSU, President of CVDA, and Senior Agriculture Attaché of the US Embassy in Beijing also attended this workshop. Photo courtesy of Ms. Li Zhang of the Chinese Veterinary Drug Association.

The rapid spread of ASF in the past four years indicates that current prevention and control measures are not effective. To improve the strategies for the prevention and control of ASF, the U.S.-China Center for Animal Health (USCCAH) at Kansas State University (KSU) in Manhattan, KS, United States (U.S.), and the Chinese Veterinary Drug Association (CVDA) in China jointly organized the 2023 International African Swine Fever Workshop (IASFW) on 18–20 September 2023, in Beijing, China (Figure 2). This workshop was sponsored by the United States Department of Agriculture Foreign Agriculture Service (USDA-FAS), Harbin Veterinary Research Institute in China, and Zoetis Inc. The objective of this workshop was to provide a platform for prominent scientists who work on the subject of ASF to unite and (i) share their knowledge and expertise; (ii) share lessons and experiences learned from China and other countries; (iii) identify knowledge gaps; (iv) foster ASF research collaborations among meeting attendees in areas of ASF vaccine development, diagnostic tool evaluation, epidemiology, and training.

## 2. Workshop Sessions and Highlights

This workshop started with an opening ceremony and remarks from invited leaders including Jishu Shi (Director of USCCAH at KSU), Xuepeng Cai (President, CVDA), David Rosowsky (Vice President for Research, KSU), Zhigao Bu (Director General of Harbin Veterinary Research Institute, Chinese Academy of Agricultural Sciences), Adam Michael Branson (Senior Agriculture Attaché, USDA-FAS at US Embassy in Beijing), Ashley Elizabeth Van Batavia (Agriculture Attaché, USDA-APHIS at US Embassy in Beijing), and Kewen Wang (Director, Technical Services of Swine Business in China, Zoetis). After the opening ceremony and two keynote lectures (Dixon, Bu; see below), four scientific sessions followed with presentations and panel discussions concerning ASF live attenuated vaccines (Session #1), subunit vaccines and mechanisms of protective immunity (Session #2), ASF pathogenesis and diagnostics (Session #3), and epidemiology and ASF control on swine farms (Session #4).

### 2.1. Keynote Lectures (Moderator: Jishu Shi)

Linda Dixon reported the recent advances and knowledge gaps in ASF research, including the structure of the African swine fever virus (ASFV), ASFV–cell interactions, pathogenesis, immune responses, and vaccine development. Zhigao Bu reported the epidemiology and outbreaks of ASFV variants in China, especially the spreading of a novel recombinant ASFV representing a mixture of both genotype I (GI) and type II (GII) viruses [16].

Panel Discussions and Highlights:

(i) More studies on ASFV proteins/complexes and ASFV–cell interactions are needed to understand ASFV replication and assembly further and find targets for antibodies and antivirals.
(ii) For modified live attenuated vaccines (LAVs), a better understanding of protective immunity, further safety and efficacy testing, the ability to differentiate infected from vaccinated animals (DIVA), permanent cell lines for LAV production, and mathematical modeling and experimental testing to determine vaccine coverage is needed.
(iii) For subunit vaccines, further research on antigens to refine pools required, mechanisms of protection, and testing additional vectors/delivery methods (mRNAs?) are needed.
(iv) We should consider alternative strategies for ASF prevention and control, such as single replication cycle vaccines using helper cell lines and gene-edited resistant pigs.
(v) Considering the rapid spread of the GI-GII recombinant ASFV in China (with possible spread to neighboring countries), comprehensive surveillance, rapid diagnostic tools, and specific control measures for the GI-GII recombinant ASFV are urgently needed.

### 2.2. Session #1: ASF Live Attenuated Vaccines (Moderator: Jishu Shi)

Dongming Zhao provided an update on ASF LAV research in China. Notably, previously described genotype II LAVs did not induce protection against the GI-GII recombinant ASFV strains circulating in some regions in China [16,17]. Linda Dixon presented her research on developing multi-gene deleted ASF LAVs. This included replacing the wild-type gene for the CD2v protein with a mutant version that retained cell surface expression but did not bind to red blood cells to reduce virus persistence in the blood [18]. Quang Lam Truong reported the development and evaluation of an LAV candidate in Vietnam [19]. Lihua Wang reported the immune responses associated with a safe and efficacious LAV candidate called KNB-LAV1 [19].

Panel Discussions and Highlights:

(i) Safety, efficacy, and genetic stability of current candidate ASF LAVs require comprehensive clinical evaluations prior to country-wide field application.
(ii) Current multi-gene deleted ASFV GII-based LAVs cannot protect pigs against challenge with the GI-GII recombinant ASFV strains circulating in China.
(iii) Cross-protection from different ASFV genotypes (heterologous immunity) combined with a stable, permanent cell line for manufacturing LAVs, as well as the development of DIVA LAVs, are critical issues that need to be resolved.
(iv) LAVs generated by passaging in unnatural hosts or cell lines have proven to be an effective means for preventing many human/animal viral diseases; the successful generation of ASF LAVs through cell passage by a joint effort of researchers at VNUA and KSU indicates that more attention should be given to ASF LAVs developed by cell passage.
(v) A novel virus neutralization test (VNT) method that does not depend on porcine macrophage cell lines should be developed to evaluate the immune responses after vaccination.
(vi) In addition to IFN-gamma ELISpot (enzyme-linked immunosorbent spot) assays, we should develop more assays to test the cellular immune responses for ASF antigens/vaccines, such as assays to measure the induction and specificity of ASFV-specific T-cells.

### 2.3. Session #2: ASF Subunit Vaccines and Mechanisms of Protective Immunity (Moderator: Dongming Zhao)

Jürgen A. Richt presented research on recombinant and vector-based ASF subunit vaccines [20]. Meilin Jin reported anti-ASFV therapeutics based on active functional small molecules and mucosal defense [21]. Christopher Netherton reported on the discovery of ASFV antigens important for subunit vaccine development [22]. Charaf Benarafa described a detailed analysis of host immune responses against ASFV and presented innate and adaptive transcriptomic profiles in the blood of ASFV-infected pigs that were associated with resilience to disease and protective adaptive response [23].

Panel Discussions and Highlights:

(i) Antibodies are not thought to be 100% efficient in protecting pigs from ASFV challenge. Neutralizing antibodies are inconsistently detected. Mechanisms/correlates of protection are still unknown. A better understanding of the mechanisms of protection of LAVs may guide the development of subunit vaccines.
(ii) Subunit vaccines can induce enhancement of ASF disease, most likely due to antibody-dependent enhancement (ADE) as reported for many macrophage-tropic pathogens.
(iii) Host immune status contributes to ASF disease severity following infection with the attenuated Estonia 2014 strain [23]. Although infection with a virulent ASFV strain is lethal for both SPF (specific pathogen-free) and conventional farm pigs, SPF pigs show higher resilience to disease after inoculation with an attenuated (Estonia 2014) ASFV strain compared to farm pigs. This phenomenon may impact the safety of LAV.
(iv) SPF pigs show complete resistance to ASFV Armenia 2008 challenge after recovery from Estonia 2014 infection. Protective immunity correlated with innate and adaptive immune responses in the blood, as shown by transcriptomic analyses. Studies of SPF pigs may help identify key correlates of protection when evaluating ASF vaccine candidates.
(v) Active functional small molecules showed anti-ASFV activities and could protect pigs against virulent ASFV challenge [21]. However, studies on antiviral mechanisms, the structure of the molecules, antimicrobial resistance, and dose- and time-dependent effectiveness, i.e., a more comprehensive evaluation of these small molecules is needed.

### 2.4. Session #3: ASF Pathogenesis and Diagnostics (Moderator: Charaf Benarafa)

Llilianne Ganges reported the development and application of new diagnostic tools for ASFV using minimal equipment [24]. Changjiang Weng presented a summary on the “tricks” that ASFV uses to escape host anti-viral innate immune responses [25,26]. Guiqing Peng reported on the inhibition of host gene expression by ASFV and its application in ASF vaccine development. Ana Reis reported on the identification and characterization of ASFV non-essential genes for rational design of LAVs [27]. Jun Han reported a new mechanism of ASFV entry into porcine macrophages using apoptotic bodies [28].

Panel Discussions and Highlights:

(i) Loop-mediated isothermal amplification (LAMP) with high sensitivity and specificity to amplify efficiently ASFV DNA under isothermal conditions is a field friendly detection method for ASFV. This assay could be used in remote locations, can be adapted to be performed using minimal equipment and with minimally invasive samples. The use of LAMP and other point-of-care diagnostic tests could prove to be a useful alternative for the early detection of ASFV, lending itself to faster action, having an overall positive impact to support the ASF control. Official regulations and guidelines for the use of point-of-care diagnostic tests in surveillance and control programs for notifiable diseases for the WOAH need to be established.
(ii) The mechanisms of ASFV immune evasion are not fully understood. More basic research studies are still needed. These include studies to determine how ASFV infection induces immune responses and modulates inflammatory responses and cell death. What are the genes/proteins related to ASFV virulence/protection?
(iii) Targets for ASFV attenuation may include interferon (IFN) inhibitors, cell adhesion molecules (such as CD2v), and inhibitors of programmed cell death. Safe and efficacious ASF LAV strains will most likely have multiple genes deleted to obtain a balance between virus replication and induction of immunity.

### 2.5. Session #4: Epidemiology and ASF Control on Swine Farms (Moderator: Llilianne Ganges)

Mary-Louise Penrith discussed her experiences in ASF control measures in endemic countries in Africa [29,30,31,32]. Yupeng Mo presented the lessons learned in ASF prevention and control in large swine production systems in China. Zhipeng Su reported on diagnostic control measures, including on-farm PCR for ASF control in large swine operations in China. Dang Vu Hoang presented data on the molecular epidemiology of ASFV strains circulating in Vietnam. Roman Pogranichniy discussed the importance of biosecurity on swine farms in ASF-endemic areas such as Ukraine [33] and Vietnam. Adrian Balaban presented lessons learned after 6 years of fighting ASF outbreaks and spread in Romania.

Panel Discussions and Highlights:

(i) Currently, our strongest weapon against ASF is good/enhanced biosecurity. Certain ASF features, such as ASFV is sensitive to heat, detergents, and a wide range of disinfectants; ASFV only survives in the environment when protected by organic material; and aerosols are only effective over distances of a few meters in an enclosed space, clearly indicate that good biosecurity measures can protect pigs from ASFV infection. Confining pigs, restricting access to them, providing dedicated and thoroughly disinfected footwear, assuring that newly introduced pigs are from “safe” sources, and ensuring the safety of feed will prevent an ASFV incursion.
(ii) Prevention and control of ASF needs to involve all actors in the pig value chain, including various stakeholders, animal health service providers, communities in which pigs are kept, and farmer-based surveillance for early detection and reporting.
(iii) In ASF-endemic areas, farmers raising pigs free of ASF need to strictly implement biosecurity measures on their farms, use ASF vaccines if licensed and available, and use point-of-care ASFV antigen diagnostics.
(iv) Human factors (human behavior) should always be taken into consideration in science-based recommendations/policies for ASF control and prevention since a significant part of ASFV spread is based on anthropogenic factors.

## 3. Critical Issues That Need to Be Addressed for ASF Control and Prevention in the Future

### 3.1. Know the Enemy through Supporting Basic and Innovative Research

ASF prevention and control is a worldwide challenge. There have not been many significant advances in ASF control and prevention since it was first reported approximately 100 years ago. Although the last five years witnessed substantial growth in ASF research, critical research gaps remain. They include details on the complex ASFV virion structure and the role and function of individual ASFV genes (ASFV has a large genome with 80% of its approximately 150 genes functionally unknown) as well as detailed studies on the mechanisms of ASFV entry, replication, immune regulation, and protective immunity. These gaps are the limiting factors in our knowledge of ASFV molecular biology and viral pathogenesis. 

As Sun Tzu said in his famous book “The Art of War”: “If you know the enemy and know yourself, you need not fear the result of battles” [34]. Therefore, government/federal agencies, philanthropic entities, and the veterinary pharmaceutical industry need to invest seriously in basic ASFV research. Critical research areas to invest in include ASFV-host interaction, epidemiology, pathogenesis, immune responses, safe and efficacious live and subunit vaccines, DIVA vaccines, point-of-contact diagnostics, swine farm biosafety, biosecurity risk management systems, and farmer-based surveillance systems for early detection and reporting.

### 3.2. Develop Science-Based ASF Outbreak Emergency Management Policies

All actors of the pig value chain, including governments (state and federal levels), swine farmers, pork processing plants, animal health service providers, and communities in which pigs are kept, should participate in and support the development and implementation of science-based ASF outbreak emergency management policies. Critical elements of the policies should include: (i) ASFV surveillance (may need to be at the level of a pig pen or cohort of pig pens): rapid and accurate identification of ASF cases, infected premises, and contact premises, as well as investigate the source of the outbreak; (ii) Science-based cleaning and disinfection measures, quarantine, and movement control measures; (iii) Proper disposal of a large amount of animal carcasses and contaminated materials (e.g., bedding, feed): this needs to be done in a short period of time in a restricted area; (iv) Indemnity payments: efficient communication between actors in the pig value chain to receive adequate compensation for animal losses as quickly as possible; (v) Effective communication locally, regionally, and nationally to minimize public panic and fear, and address rumors, inaccuracies, and misperceptions.

### 3.3. Collaborative Approach

A collaborative approach is the best way forward for ASF control and eradication. ASF outbreaks affect many parts of society, including swine producers, workers on the farm, grain and feed producers, pork processing plants, grocery stores, truck drivers, animal health companies, restaurants, international and national pork/grain/feed importers and exporters, and all consumers of pork products. The swine industry and governments should work together to confront this critical threat [35]. Governments should ensure their goals and policies to control ASF are fully supported by all actors in the pig value chain, including swine farm owners, farm employees, pork processing plants, animal health companies, veterinarians, regulatory agencies, social media, and the public. The pork industry must make biosecurity a top priority. Only a licensed and authorized safe and efficacious ASF vaccine should be used. Understanding and obeying the rule that “ASFV positive products should not be produced, transported, sold, or consumed by anyone” by all stakeholders is critical in the collaborative approach to confronting, defeating, and controlling ASF (the enemy).

## 4. Conclusions

The 2023 International African Swine Fever Workshop was very successful, given that it brought together several of the most active ASF scientists in the world. It facilitated a scientific exchange with lessons learned and experiences that provided the basis for discussions on critical issues that need to be resolved for current and future ASF control and prevention. In addition, it allowed the creation of a scientific platform to review ongoing scientific collaborations and establish new ones focusing on research on the structural and non-structural components of the ASF virion and its complex host cell interactions. Likewise, the importance of establishing standard criteria for the development of diagnostic methods and vaccines was discussed, and the need to standardize and homogenize these standard criteria globally to evaluate the effectiveness of ASFvaccines and the specificity/sensitivity of diagnostic tools for ASFV was emphasized.

Lively panel discussions after each session’s presentations were highly enjoyed by the audience. We hope that the session summaries, highlights, and discussions provided in this report will encourage interested researchers to join us at the **2024 International African Swine Fever Workshop**, which is presently being planned to take place in Kansas, United States. More information can be obtained from the conference host, Dr Jishu Shi, jshi@vet.k-state.edu.

## Figures and Tables

**Table 1:** Number of outbreaks, cases, and animal losses caused by ASF in the different world regions, January 2021 to August 2023 (Source: Situation reports for African swine fever of WOAH, https://www.woah.org/en/disease/african-swine-fever/#ui-id-2, accessed on 10 December 2023).

	Outbreaks		Cases		Losses *
	Domestic Pigs	Wild Boar	Domestic Pigs	Wild Boar	Domestic Pigs
Africa	212	0	22,786	0	24,321
Americas	278	0	9957	0	18,857
Asia	918	1284	68,339	1962	388,035
Europe	3815	16,214	85,2500	26,716	1,076,951
Oceania	0	0	0	0	0
Total	5229	17,498	953,582	28,678	1,508,164

* Losses (deaths + animals killed and disposed of): refers to losses in swine farms affected by the outbreaks; it does not include the animals culled in areas around the outbreak for controlling the disease.

## Data Availability

All relevant data to support the descriptions and discussions in the text are available from authors and corresponding authors upon reasonable request.
